# Extrapulmonary Small Cell Carcinoma of the Anal Canal: A Case Report and Review of the Literature

**DOI:** 10.1155/2012/341432

**Published:** 2012-02-20

**Authors:** Joshua M. Eberhardt, Karen Brown, Shelly Lo, Suneel Nagda, Sherri Yong

**Affiliations:** ^1^Department of Surgery, Section of Colorectal Surgery, Loyola University Medical Center, 2160 S First Avenue, Maywood, IL 60153, USA; ^2^Department of Medical Oncology, Loyola University Medical Center, 2160 S First Avenue, Maywood, IL 60153, USA; ^3^Department of Radiation Oncology, Loyola University Medical Center, 2160 S First Avenue, Maywood, IL 60153, USA; ^4^Department of Pathology, Loyola University Medical Center, 2160 S First Avenue, Maywood, IL 60153, USA

## Abstract

*Purpose*. Extrapulmonary small cell carcinoma affecting the anal canal is a rare and poorly understood entity which can, in its early stages, masquerade as benign anorectal disease such as hemorrhoids. *Methods*. We report a case of this rare malignancy which initially presented with hematochezia and anal pain. We also review the literature with regard to previously described cases and management strategies including the role of surgery. *Results*. Despite aggressive multidisciplinary treatment consisting of chemotherapy and radiation, the disease progressed rapidly with dissemination occurring only three months after completion of treatment. Because of the aggressive nature of this tumor, the treatment options for this almost universally fatal malignancy are often palliative in nature. *Conclusion*. Chemoradiotherapy is likely the most reasonable approach to extrapulmonary small cell carcinoma of the anal canal given its aggressiveness.

## 1. Introduction

Anal carcinomas represent approximately 4% of all gastrointestinal cancers diagnosed annually in the United States with an estimated 5260 new cases to be found in 2010 [1]. Of those, the majority are squamous carcinomas for which the treatment is well established and the prognosis usually favorable [2]. Extrapulmonary small cell carcinoma can also arise in the anal canal. Although it is rare, it is an important clinical entity due to its aggressive course and lack of well-defined and standardized treatment. Here we report a case of small cell carcinoma of the anal canal including our management and a review of the literature.

## 2. Case Report

**Figure 1:**
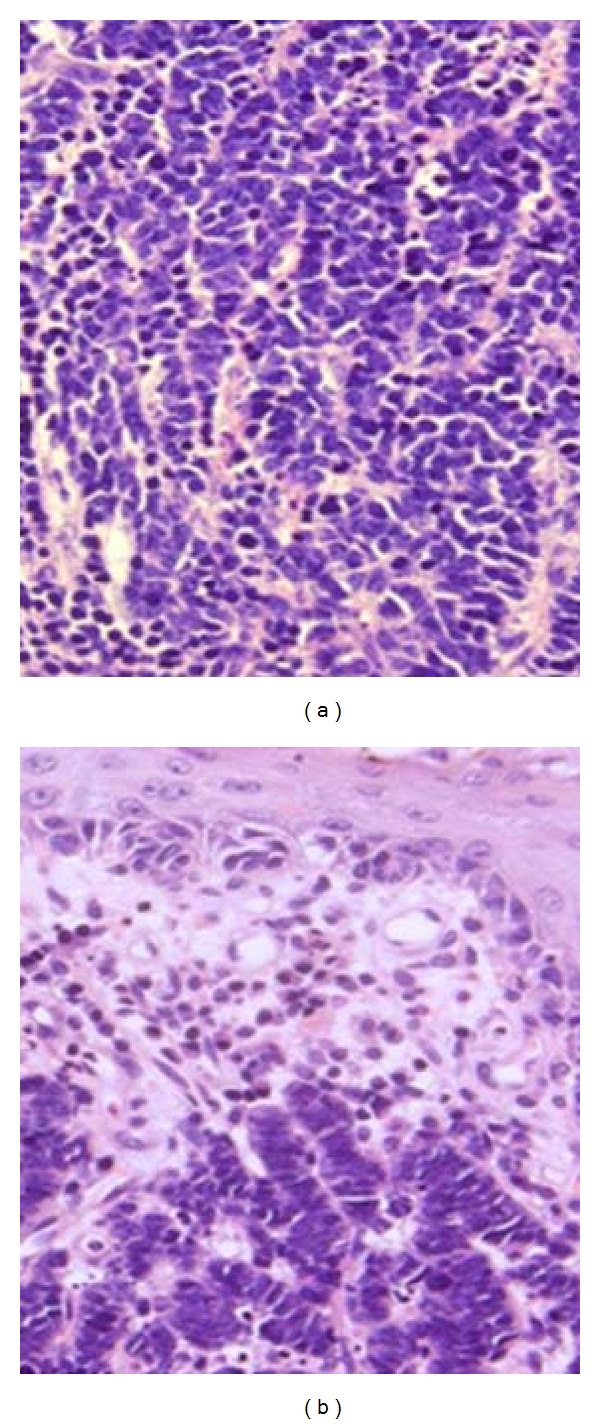
Histologic section of the inguinal lymph node (a) and anal canal lesion (b) demonstrating small to intermediate malignant cells with little cytoplasm, nuclear molding, necrosis, and apoptosis. Both showed positive immunoreactivity for keratin AE1/3, synaptophysin and were negative for p63 (H&E 400x).

A 63-year-old female developed episodic hematochezia and anal pain. Initial anorectal exam revealed only small internal hemorrhoids and colonoscopy demonstrated no proximal pathology. Weeks later she developed an enlarged, symptomatic right inguinal lymph node which was excised and revealed metastatic small cell carcinoma. She was then referred to us for evaluation. At this time digital exam revealed a 2 cm fixed mass in the anal canal at the level of the dentate line. Biopsy of the anal canal mass demonstrated small cell carcinoma which was histologically similar to that seen in the inguinal node (Figure 1).

**Figure 2:**
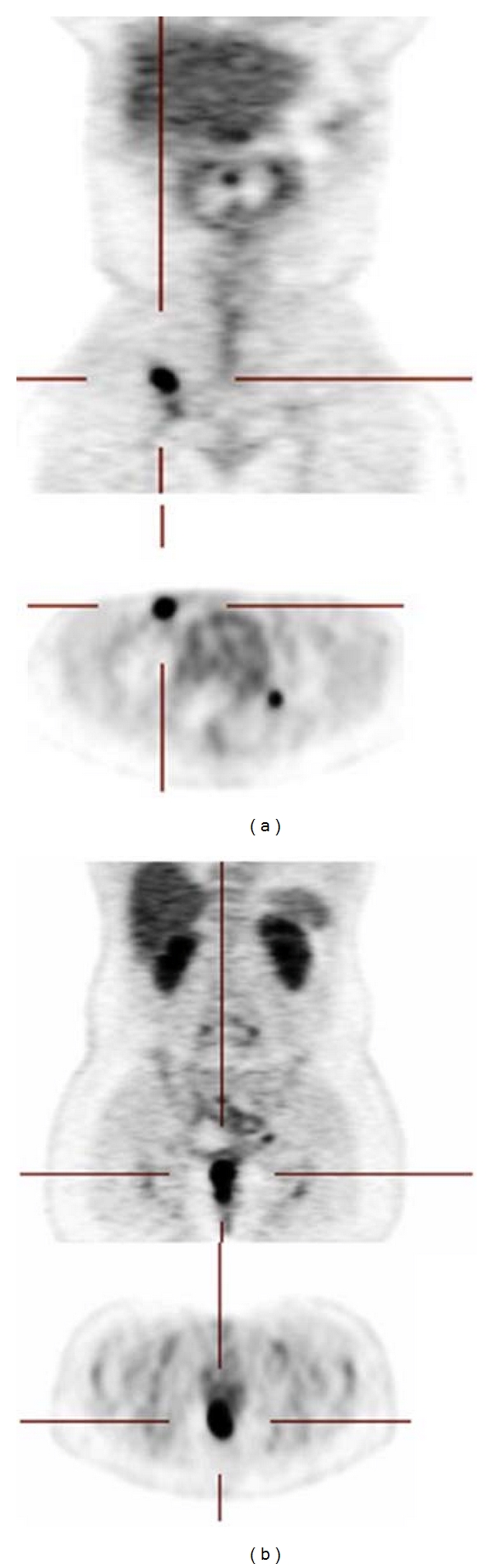
PET scan showing uptake in right inguinal region (a) and anal canal (b).

Staging CT of the chest, abdomen, and pelvis, and MRI of the brain did not reveal distant metastatic disease nor a primary lung tumor. 18F-FDG PET scan demonstrated uptake in the anal canal and right inguinal region (Figure 2). Routine laboratory parameters were normal and serology was negative for HIV infection. She was treated with chemoradiotherapy consisting of cisplatin and etoposide concurrent with radiation therapy during cycles 2 and 3. She received a total dose of 54 Gy in 27 fractions to the gross anal tumor and the right inguinal region and 47 Gy in 27 fractions to the pelvis. Intensity-modulated radiation therapy was used.

**Figure 3:**
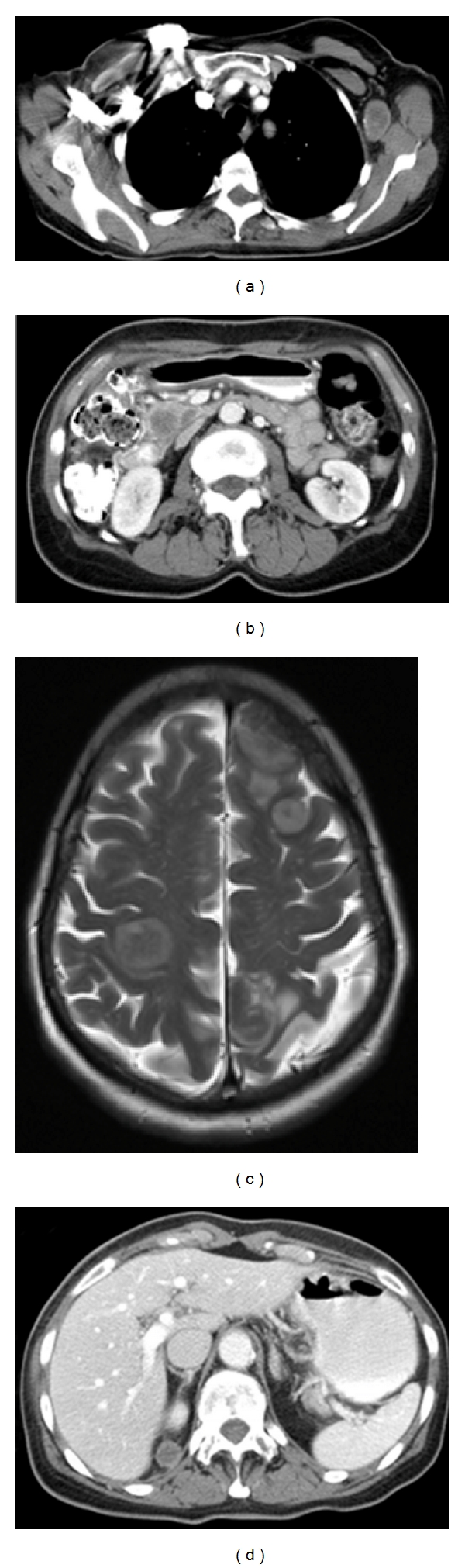
Metastatic disease: (clockwise starting from upper left) left axillary lymph node, pancreatic lesions, multiple brain metastases, and right superior perinephric mass.

Her anal symptoms improved markedly during the first few weeks of treatment. Clinical examination four weeks after completion of chemoradiotherapy revealed complete regression of the anal tumor and no palpable recurrence in the inguinal regions. However, three months after the completion of chemoradiotherapy, she developed new onset arm weakness. Imaging revealed the development of extensive brain and intra-abdominal metastasis. Pancreas, adrenal gland, liver, breast, lung, brain, and lymph nodes all showed evidence of disease on imaging (Figure 3). At this point, after multiple discussions with the patient and family, we proceeded with palliative measures and she ultimately expired 10 months after the diagnosis was made.

## 3. Discussion

Small cell lung carcinoma comprises about 13% of all lung cancers [3]. Histologically identical neoplasms found outside of the lung and with no evidence of pulmonary involvement are termed extrapulmonary small cell carcinomas (EPSCCs). Regardless of where it occurs, EPSCC is rare and has a reported incidence in the US of 0.1 to 0.4% [4]. Approximately 650 cases of gastrointestinal EPSCC have been reported in the literature and the majority of these have been located in the esophagus, colon, and rectum [5]. Colorectal EPSCC is thought to occur with an incidence of <1% of all colorectal malignancies [6]. In fact, a review of 50 years of malignant colorectal pathology at the Mayo Clinic found the incidence of EPSCC to be 2 per 1000 malignant lesions of the bowel [7].

Small cell carcinoma of the anal canal is extremely rare and its true incidence is not entirely known; most of the literature on this topic is in the form of isolated case reports. Additionally, the existing literature may be misleading because some reports detailing cases of small cell carcinoma affecting the rectum might have actually been describing lesions that arose in the anal canal if strict definitions of the surgical anal canal were used. The largest number of cases was reported by Boman et al.; their retrospective review of 188 cases of anal canal carcinomas seen over a 27-year period found that only 13 of them were actually small cell carcinoma [8].  Table 1 lists the case reports and series of anal canal EPSCC reported in the English literature where specific information with regard to treatment and outcome was provided.

Because of the rarity of EPSCC, not much is known about its origin. Some reports refer to these carcinomas as “neuroendocrine,” but others feel that this terminology implies an embryologic origin that, as of yet, is still unproven [13]. Nonetheless, three theories have been purported to explain the origins of EPSCC: enteric neuroendocrine cells derived from the neural crest, pluripotent stem cells derived from endoderm, or from cells that simply arise out of normal organ-specific cancers when certain clones reach the end stages of genetic change [14–17]. The exact histogenesis is controversial; however, the second and third theories seem attractive because they provide a reason for why EPSCC of the GI tract often contains various degrees of other epithelial cell types [18]. For example, many reports of EPSCC arising throughout the GI tract describe the lesions occurring next to or within adenomas [18, 19].

It is not known if any acquired or life-style-related factors predispose to the development of EPSCC. Although our patient was a long-time smoker and smoking is frequently associated with SCLC, approximately 30% of patients with EPSCC are nonsmokers [20]. While radiation has been implicated in SCLC and there is a case report of a rectal EPSCC developing in a patient 12 years after pelvic radiation for a gynecologic malignancy, an association with EPSCC is not proven [21, 22]. We know that the immune suppression resulting from HIV infection and AIDS can increase the risk of developing anal squamous carcinoma. Although our patient was HIV negative, some of the previous case reports of anal EPSCC occurred in HIV-infected individuals (Table 1) thus causing speculation as to the role of this infection in the development of this rare cancer [9, 11]. Given the small number of patients it is impossible to know if there is an association.

As highlighted by our case, the initial staging workup for suspected EPSCC consists of imaging the brain, chest, abdomen, and pelvis to rule out the lung as the primary and assess for distant or locoregional disease. Given the lymphatic drainage of the anal canal, if this is the primary location special attention must be paid to the inguinal lymph nodes. Suspicious lymphadenopathy not amenable to biopsy should be further investigated via PET scan as they are typically PET avid. Since our case presented with locoregional disease only, we proceeded with treatment similar to that which is done for limited stage SCLC where the typical regimen is concurrent chemoradiotherapy using cisplatin and etoposide. Although the patient's anal symptoms improved drastically and the primary tumor appeared to have completely regressed by 4 weeks after chemoradiotherapy, significant distant disease arose shortly thereafter.

Unfortunately, no well-studied treatment guidelines exist for anal EPSCC; thus our current strategy relies on how it is treated when it occurs in other anatomic locations. For example, Hoskins et al. found that of 34 patients with EPSCC of the cervix treated with chemoradiotherapy, 55% were long-term survivors [23]. Although there are few patients reported on in the literature, it seems that this result has not been duplicated in cases of anal EPSCC. Of those that have been treated with chemoradiotherapy, most have had at least an initial response to treatment but, as in our case, this was quickly followed by emergence of disease in other locations (Table 1) [9, 10, 12]. An explanation for this phenomenon, as outlined by Brenner et al. might lay in the fact that these tumors often contain an admixture of squamous or adenocarcinoma; when the lung-type therapy is used, the subsequent recurrence might be due to the proliferation of nonsmall cell clones [17].

Surgery has been employed in cases of anal EPSCC but the results have been equally disappointing. It did not significantly impact the progression of disease in the case reported by Nakahara et al. [11]. Moreover, of the 7 anal canal EPSCC patients treated with APR in the largest report, six developed recurrence at a median interval of 4 months from surgery and died of disease roughly 2 months thereafter [8]. The authors felt that surgery was a “gross failure” [8]. However, one of the seven did survive more than 5 years without recurrence and this was the only patient in their series where the tumor was pathologically determined to be “confined to the anal epithelium and subepithelial connective tissue” [8]. By extrapolation it seems that this would be a T1 tumor by today's standards. Based on this single case the question that is raised is as follows: should a patient with an anal canal EPSCC determined by EUS to be T1 and by imaging to have no distant metastatic disease be offered radical resection in the form of APR? There simply is not enough evidence to guide decision but it is possible that this is the only scenario in which surgery can offer more than palliation alone.

## 4. Conclusion

EPSCC of the anal canal is rare, and in its early stages may have a clinical presentation similar to other routine anorectal problems. It continues to be associated with a very poor prognosis, and at this point, treatment with chemoradiotherapy seems to be the most reasonable. The rapid distant dissemination occurring in our patient despite treatment highlights the fact that this disease is very aggressive and further investigation must be done to define novel therapeutic options.

## Figures and Tables

**Table 1:** Summary of reported cases of anal canal EPSCC.

Author (yr)	*n*	Demographic	Metastatic disease at presentation	Treatment	Clinical course
Alcindor et al. (2008) [9]	1	45y M HIV+	y	Chemoradiation, C/E, G-CSF	Primary responded, distant disease progressed, died 6 mo after dx
Doddi et al. (2009) [10]	1	60y F	n	Chemoradiation, C/E	Primary responded, distant disease developed, died 18 mo after dx
Nakahara et al. (1993) [11]	1	48y M HIV+	n	radiation, APR, chemoradiation, C/E, G-CSF	Pelvic local recurrence after surgery, distant disease developed, patient committed suicide 3 mo after surgery
Meyer et al. (2007) [12]	1	41y F	y	Chemoradiation, C/E	Partial response of primary, complete response of liver and lung metastasis, but new bone metastasis developed, died 10 mo after dx
Boman et al. (1983) [8]	13	ns	5y	ns	Median survival 2 mo after dx
			8n	7 APR	6 patients recurred and had median survival of 6 mo after surgery 1 patient with 5 yr survival
				1 radiation	Died 8 mo after treatment

Ns: not specified; APR: abdominoperineal resection; C/E: cisplatin and etoposide; G-CSF: granulocyte colony stimulating factor.
